# Artificial Intelligence in Psychiatry: A Comprehensive Literature Review

**DOI:** 10.1192/j.eurpsy.2024.173

**Published:** 2024-08-27

**Authors:** M. Gerantia

**Affiliations:** Addiction, Center of Mental Health and addiction prevention, Tbilisi, Georgia

## Abstract

**Introduction:**

The incorporation of artificial intelligence (AI) in healthcare, especially in mental health services, offers potential advancements in efficiency and personalization. As AI technologies like machine learning and natural language processing (NLP) continue to evolve, it’s vital to evaluate their applications in psychiatry comprehensively.

**Objectives:**

This review aims to summarize and characterize studies that used AI, particularly machine learning and NLP, in mental health. Additionally, it endeavors to understand how these technologies may enhance diagnostic tools, symptom monitoring, and delivery of personalized treatment in psychiatry.

**Methods:**

Adhering to PRISMA guidelines, a systematic search was executed across multiple medical databases, including PubMed, Scopus, ScienceDirect, and PsycINFO. Keywords encompassed machine learning, data mining, psychiatry, and mental health. Exclusion criteria included non-English papers, anonymization process descriptions, case studies, conference papers, and other reviews. Data from various segments in the provided information were synthesized to capture the broader picture of AI’s application in psychiatry.

**Results:**

From the 327 articles initially identified, 58 were chosen for detailed review. Studies predominantly revolved around three main populations: patients in medical databases, emergency room visitors, and social media users. The primary applications of AI entailed symptom extraction, illness severity classification, therapy effectiveness comparison, and psychopathological insights derivation. Data sources mainly included medical records and social media, with Python emerging as the preferred platform for most studies.

**Conclusions:**

While AI shows immense promise in revolutionizing mental health care, its current applications largely confirm existing clinical hypotheses. Ethical concerns, such as patient privacy and data biases, remain paramount. Future work should delve deeper into these challenges while further exploring AI’s potential in clinical psychiatry practice.

**Disclosure of Interest:**

None Declared

